# Proteomic View of the Crosstalk between *Lactobacillus mucosae* and Intestinal Epithelial Cells in Co-culture Revealed by Q Exactive-Based Quantitative Proteomics

**DOI:** 10.3389/fmicb.2017.02459

**Published:** 2017-12-12

**Authors:** Edward Alain B. Pajarillo, Sang Hoon Kim, Valerie Diane Valeriano, Ji Yoon Lee, Dae-Kyung Kang

**Affiliations:** ^1^Department of Animal Resources Science, Dankook University, Cheonan, South Korea; ^2^National Instrumentation Center for Environmental Management, Seoul National University, Seoul, South Korea

**Keywords:** *Lactobacillus mucosae*, adhesion, host–microbe interaction, porcine intestinal epithelial cells, label-free proteomics

## Abstract

*Lactobacilli* are bacteria that are beneficial to host health, but information on communication between *Lactobacilli* and host cells in the intestine is lacking. In this study, we examined the proteomes of the *Lactobacillus mucosae* strain LM1, as a model of beneficial bacteria, and the intestinal porcine epithelial cell line (IPEC-J2) after co-culture. Label-free proteomics demonstrated the high-throughput capability of the technique, and robust characterization of the functional profiles and changes in the bacteria and intestinal cells was achieved in pure and mixed cultures. After co-culture, we identified totals of 376 and 653 differentially expressed proteins in the LM1 and IPEC-J2 proteomes, respectively. The major proteomic changes in the LM1 strain occurred in the functional categories of transcription, general function, and translation, whereas those in IPEC-J2 cells involved metabolic and cellular processes, and cellular component organization/biogenesis. Among them, elongation factor Tu, glyceraldehyde 3-phosphate dehydrogenase, and phosphocarrier protein HPr, which are known to be involved in bacterial adhesion, were upregulated in LM1. In contrast, proteins involved in tight junction assembly, actin organization, and genetic information processing (i.e., histones and signaling pathways) were significantly upregulated in IPEC-J2 cells. Furthermore, we identified functional pathways that are possibly involved in host–microbe crosstalk and response. These findings will provide novel insights into host–bacteria communication and the molecular mechanism of probiotic establishment in the intestine.

## Introduction

The microenvironment of the gastrointestinal tract of mammals serves as a niche to trillions of allochthonous or autochthonous microorganisms that express various functional genes and persist in the host. *Lactobacillus* and *Bifidobacterium* are gut commensal bacteria that are potentially beneficial to the host (Bottazzi, 1988; Marteau et al., 1992). *Lactobacillus mucosae* is a natural resident of porcine, bovine, and human intestinal mucosa (Roos et al., 2000; Lee et al., 2012; Bleckwedel et al., 2014; Ryan et al., 2015). *L. mucosae*, which has mucus-binding activity, was first identified in pig intestines and is closely related to *L. reuteri* (Roos et al., 2000). Since then, various assays and characterization studies have shown that *L. mucosae* strains have the potential to be probiotic. In particular, *L. mucosae* strains might help improve mucosal immunity and pathogen resistance by increasing epithelial impermeability and barrier function (Valeriano et al., 2014), and producing secondary metabolites (Pajarillo et al., 2015) and antimicrobial compounds (Drissi et al., 2015). Genomic, proteomic, and biochemical profiling studies on *L. mucosae* strains also revealed potential probiotic activity and the presence of pathways involved in the biosynthesis of exopolysaccharides, glycogen, succinate, and folate (London et al., 2014; Pajarillo et al., 2015).

IPEC-J2 cells are a well-maintained cell line from porcine IECs from neonatal jejunum that is widely used as an *in vitro* intestinal model for adhesion and infection studies (Skjolaas et al., 2007). Bacterial adhesion plays an important role in intestinal colonization and establishment and occurs before the stimulation of cellular activities and immune response from IECs (Van Tassell and Miller, 2011; Juge, 2012). The LM1 strain showed strong adhesion to crude mucus, mucin, and IPEC-J2 cells in our previous report (Valeriano et al., 2014, 2016). Despite the plethora of studies on adhesion ability, this ability is particularly challenging to study due to undetermined species- and strain-specific factors, and varying experimental conditions. In lactobacilli studies, bacteria-specific components have been identified that exhibit possible signaling or effector ability in certain gastrointestinal regions (Kleerebezem et al., 2010; Górska et al., 2016); however, their cellular mechanism of action and binding targets remain to be discovered. Thus, comprehensive “omic” studies are needed to investigate the potential probiotic mechanism of LM1. Understanding these cellular mechanisms requires genomic and transcriptomic studies; however, information from these studies can often be limited. On the other hand, global proteomic studies can be used to identify post-translational modifications and the precise biological functions of the organism being studied (Chandramouli and Qian, 2009).

In this study, we employed a gel-free proteomic approach to investigate proteome changes in both LM1 and IPEC-J2 cells after co-incubation. Q Exactive Orbitrap MS was used for a full proteomic scan of bacterial and intestinal cells, and for a large-scale quantitative analysis of protein dynamics during host–microbe interaction. This is the first proteomic study to use this method to obtain novel insights into probiotic adhesion to IECs.

## Materials and Methods

### General Experimental Design

The experimental design for proteomic analysis of pure and mixed cultures used in this study is summarized in **Supplementary Figure S1**. LM1 (Taxonomy ID: 1130798) was grown statically in MRS medium (Difco, United States) at 37°C. The IPEC-J2 was grown in Dulbecco’s modified Eagle’s medium/F-12 Ham (DMEM/F12) containing 0.12% NaHCO_3_, 15 mM HEPES, pyridoxine, and L-glutamine, and (Sigma–Aldrich, United States) supplemented with 100 U/mL erythromycin, 0.5 mmol/L sodium pyruvate, and 5% fetal bovine serum (Sigma–Aldrich), and maintained in 5% CO_2_ at 37°C and 95% humidity. Cells were passaged every 3–4 days (seeding at a 1:3 ratio) and the medium was changed every other day.

Before LM1 was sub-cultured in IPEC-J2 cells, the bacterial cells were washed twice with Dulbecco’s phosphate-buffered saline (DPBS; pH 7.0; Gibco, United States). For the co-culture experiment, LM1 cells from an overnight culture were re-constituted in DMEM only (no serum, no antibiotics) to be inoculated at 1.8 × 10^8^ cells with IPEC-J2 cells as determined in a previous experiment (Pajarillo et al., 2015). Control cells of LM1 or IPEC-J2 were also incubated in DMEM only. Control and co-cultured cells were incubated in 5% CO_2_ at 37°C and 95% humidity, then cells were harvested after 1 h. For scanning electron microscopy, control and co-culture cells were washed, dehydrated with ethanol, and coated with gold. Pure and mixed cultures were examined with a scanning electron microscope (XL30CP; Philips, Netherlands). All experiments were repeated in four biological replicates.

### Protein Extraction and Quantification

Control (pure cultures of LM1 and IPEC-J2 cells) protein extracts were prepared as previously described (Pajarillo et al., 2015; Kim et al., 2016). For the co-culture (mixed culture) experiment, the bacterial cells were first harvested. To do this, the bacterial cells were aspirated from the setup, washed twice with DPBS, and then centrifuged at 15,000 *g* for 10 min at 4°C. The IPEC-J2 cells were then detached from the polystyrene 6-well plates with a sterile cell scraper. The detached IPEC-J2 cells then underwent differential centrifugation at 2500 *g* for 3 min. After discarding the medium, the collected cells were re-suspended in phosphate-buffered saline (PBS) containing a protease inhibitor cocktail and lysed by sonication. The samples were centrifuged at 15,000 *g* for 10 min and the supernatants were collected. The protein concentrations were measured by Bradford protein assay. The protein solutions were stored at -20°C until further analysis. In-solution digestion was done using trypsin (V511B, Promega, United States) and sample preparation was performed as described previously (Wiśniewski et al., 2009).

### Q Exactive MS

The quantified protein samples were analyzed for a full MS scan followed by independent analysis (IDA) MS/MS scans using a Q Exactive Orbitrap mass spectrometer (Thermo Fisher Scientific, United States) with a HESI-II. MS spectra were acquired at a resolution of 70,000 within a mass range of 350–1,800 m/z. Ion accumulation was set at a maximum injection time of 100 ms. After ion activation/dissociation, the 10 most abundant peaks (Top10 method) were measured with higher energy C-trap dissociation (HCD) at a normalized collision energy of 27% within a mass range of 100-2000 m/z. Sample fractionation was performed in solvent A (water/acetonitrile, 98:2 v/v; 0.1% formic acid). Samples were trapped with an Acclaim PepMap 100 trap column (100 μm × 2 cm, nanoViper C18, 5 μm, 100 Å) in a Dionex U 3000 RSLCnano high performance liquid chromatography (HPLC) system followed by washing with 98% solvent A for 6 min at a flow rate of 4 or 6 μL/min. The Acclaim PepMap 100 capillary column (75 μm × 15 cm, nanoViper C18, 3 μm, 100 Å) facilitated the protein separation at a flow rate of 400 nL/min. After LC, the analytical gradient was run with various percentages of solvent B in the following manner: (1) 2.0–35% for 90 min, (2) 35–95% for 10 min, (3) 90% for 5 min, and (4) 5.0% for 15 min. The resulting peptides were electro-sprayed through a coated silica tip (PicoTip emitter, New Objective) at an ion spray voltage of 2000 eV.

### Protein Identification and Annotation

All raw data files generated by MS were processed in Xcalibur Qual Browser and analyzed by Proteome Discoverer software 1.4 (Thermo Fisher Scientific, United States) and the MaxQuant program (version 1.4, Max Planck Institute, Germany) against the genomes of *L. mucosae* LM1 (GenBank accession number: NZ_CP011013.1) and *Sus scrofa* (GenBank accession number: GCA_000003025.4, annotation release 105), including the variable modifications methionine oxidation and N-terminal acetylation, and the fixed modification of carbamidomethyl cysteine. The protein sequences were downloaded from the NCBI reference sequences (RefSeq) database. Parent peptide masses and fragment masses were searched with maximal initial mass deviation of 6 and 20 ppm, respectively. Missed tryptic cleavage sited allowed was 2. All of the biological functions of the proteins from LM1 and IPEC-J2 were classified based on the most recent genome available in the NCBI database. The quantification was also performed using Proteome Discoverer software 1.4 and the MaxQuant program (Max Planck Institute, Germany), which can automatically calculate the relative abundance of peptides and the corresponding proteins. Proteins identified with two or more unique peptides were considered high confidence identifications and were used for quantification. Also, to ensure the accuracy of quantification, only proteins that had a coefficient of variation of four biological repeats of less than 20% were identified as significantly expressed proteins. The detected peptide threshold in the MaxQuant program was set to 5% false discovery rate (FDR) using an FDR-controlled algorithm called matching between runs is incorporated, which enables the MS/MS free identification of MS features in the complete data set for each single measurement.

The complete genome of *L. mucosae* LM1 was analyzed for putative secondary metabolite biosynthetic gene clusters using antiSMASH version 3.0 software using default parameters (Weber et al., 2015). Proteins with signal peptides, non-classical protein secretion properties, and a transmembrane helix structure were predicted using SignalP version 4.1 (Petersen et al., 2011), SecretomeP version 2.0a (Lonsdale et al., 2016) and TMHMM Server version 2.0 (Chen et al., 2003) softwares, respectively. Since most proteins from *S. scrofa* have high homology to the complete genomes of human (>80%) and mouse (>70%), all of the proteins from the IPEC-J2 proteome, in particular unannotated proteins, were cross-checked against the *Homo sapiens* and *Mus musculus* protein database in the NCBI and GO PANTHER database in order to identify additional proteins in the dataset.

### Bioinformatics Data Analysis

R Software (version 3.3.1, R Core Team, Australia) was used for bioinformatics and statistical analysis. In this experiment, individual proteomes of LM1 and IPEC-J2 cells were designated as control (pure culture) and co-culture/co-incubation/treatment (mixed culture). The patterns of differential protein expression were displayed by volcano plot according to the fold-change ratios. The *p*-values < 0.05 were calculated using ANOVA. Non-metric dimensional scaling (NMDS) analysis was performed for control and co-culture cells to determine the variation between individual proteomes. LEfSe was used for the quantitative analysis of biomarkers within different groups (Blankenberg et al., 2010). This technique was designed to analyze high-throughput data where the number of genes is greater than the number of samples and to provide biological class explanations to establish statistical significance, biological consistency, and effect-size estimation of predicted biomarkers. A paired student *t*-test was done to identify differences in bacterial or intestinal cell proteomes between control (C1–C4) and co-culture (T1–T4) groups. GraphPad Prism version 7 (GraphPad Software, United States) was used to create bar graphs of proteins expressed at statistically different levels.

For pathway analysis, the KEGG was used for full annotation of the functional genes by BLAST or GHOST comparisons parallel to the manually curated KEGG GENES database. The bi-directional best hit (BBH) method was used to assign the orthologs. The result contains KEGG Orthology (KO) assignments and automatically generated KEGG pathways. LM1 and IPEC-J2 proteins with a ≥4.0-fold change after co-culture were analyzed further. Proteins were analyzed using the STRING database^1^ to determine the PPI. Using the default parameters, protein networks within the LM1 or IPEC-J2 proteomes were identified at low (0.150) and medium (0.400) confidence levels, respectively. Simple tabular outputs were generated for LM1 and IPEC-J2, which were plotted in Cytoscape using its combined score and co-expression values plotted as edges (lines) and interacting proteins as nodes (circles) (Su et al., 2014). Furthermore, a correlational matrix was also generated to predict and analyze the associations and links between LM1 and IPEC-J2 proteins that had a ≥4.0-fold change after co-culture. A correlation value of 1.0 ≥*x* ≥ 0.8 for positive correlation and -1.0 ≤*x* ≤-0.8 for negative correlation was employed using the Correlational Network Analysis tool in Cytoscape. The Cytoscape tool was also used to visualize correlation networks using correlation values as edges (lines) and proteins as nodes (circles).

### Data Availability

The MS proteomics data have been deposited to the ProteomeXchange Consortium^2^ via the PRIDE partner repository with the dataset identifier PXD006851.

## Results

### Proteomic Profile of *L. mucosae* LM1 after Co-culture

**FIGURE 1:**
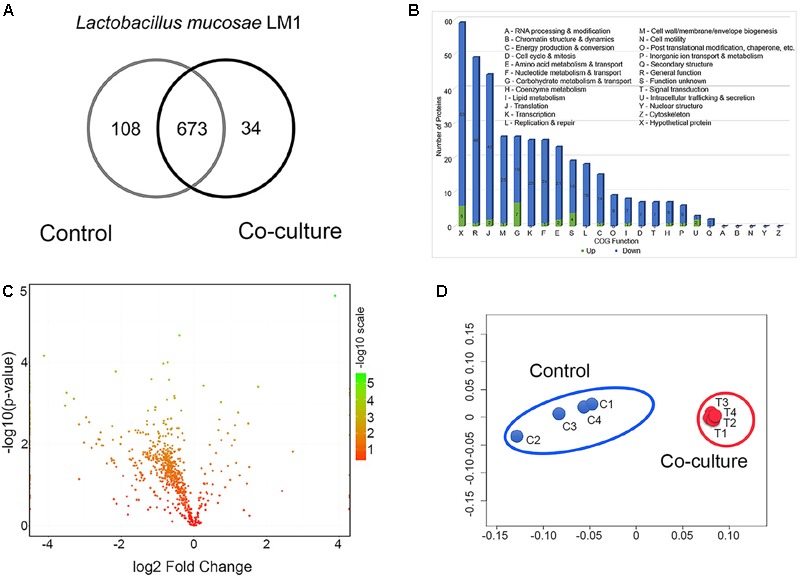
Global proteome profile and differentially expressed proteins (DEPs) from LM1 in co-culture conditions. **(A)** Venn diagram of unique and shared proteins detected from LM1. **(B)** Functional classification of LM1 proteins using Cluster of Orthologous Groups (COG). The numbers of downregulated (blue) and upregulated (green) proteins are indicated. **(C)** A volcano plot of the deactivated/downregulated (left, 348) and activated/upregulated (right, 28) proteins. *P*-values were calculated and plotted on the *y*-axis as –log10(*p*-value). Values for *y*-axis [–log10(*p*-value)] above 1.3 were considered significant. **(D)** Non-metric multidimensional scaling (NMDS) plot of individual LM1 proteomes showing distinct clustering of each group. Individual proteomes are separated in control (C1–C4) and co-culture (T1–T4).

After co-culture of *L. mucosae* LM1 with IPEC-J2 cells for 1 h, unbound LM1 cells were removed, then bound LM1 to IPEC-J2 cells were used for proteome analysis. We extracted intracellular proteins from *L. mucosae* LM1 control and after co-culture with IPEC-J2 cells to identify proteins possibly involved in cell–cell communication. Totals of 781 and 707 proteins were detected in control and after co-culture with IPEC-J2, respectively (**Figure 1A**). Almost 10% reduction in the expressed protein numbers of LM1 after co-culture might be affected by IPEC-J2 cells, for example, in nutrition competition, between the IPEC-J2 cells and LM1, with IPEC-J2 cells showing more efficient utilization of the DMEM media nutrient source, compared to the LM1 strain. However, this needs to be elucidated in the future. In addition, 673 proteins were present in both conditions, while 108 and 34 proteins were uniquely identified in control and co-culture conditions, respectively (**Figure 1A**).

The global proteins were arranged by functional categories according to their COG classification (**Figure 1B**). Proteins whose levels were either increased or decreased belonged primarily to the following categories: (i) general function, (ii) translation, (iii) cell wall/membrane/envelope biogenesis, (iv) carbohydrate metabolism and transport, (v) transcription, (vi) nucleotide metabolism and transport, and (vii) amino acid metabolism and transport (**Figure 1B**). A statistical analysis identified 300 proteins whose expression decreased from 0.83- to 0.05-fold (**Figure 1C** and Supplementary Table S1). However, 23 proteins were upregulated 1.15- to 14.7-fold in co-culture (**Figure 1C** and Supplementary Table S1). Global proteomic profiles of control and co-culture groups were compared revealing a distinct separation between the two groups (**Figure 1D**), suggesting that co-culture with IPEC-J2 induced the changes in protein expression associated with basic cellular processes and potentially adhesion ability of LM1 cells.

Based on these observations, many functional activities were reduced in co-culture conditions. This suggests that LM1 adhesion and cell–cell interaction does not require many of its basic biological and cellular processes during its establishment in the intestinal epithelia.

### *L. mucosae* LM1 Functional Proteins and Pathways Influenced by IPEC-J2 Cells

**FIGURE 2:**
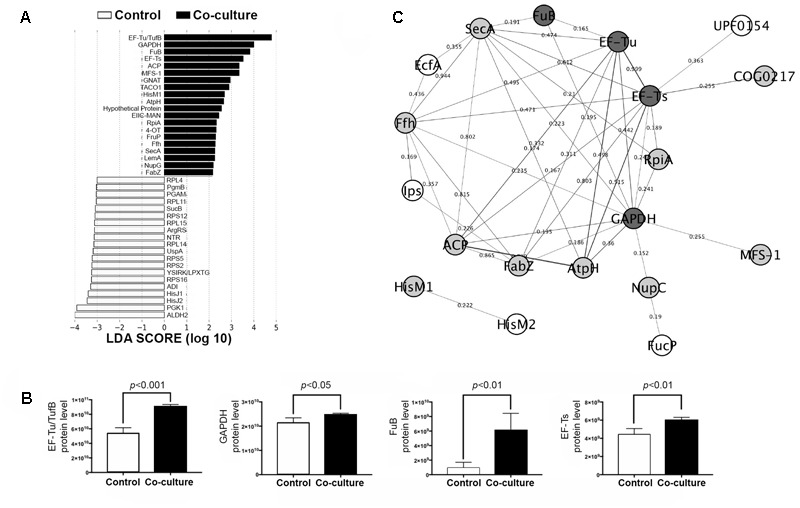
Biomarker analysis and protein–protein interaction (PPI) network in the LM1 proteome. **(A)** Linear discriminant analysis (LDA) effect size (LEfSe) score showing the statistical significance, biological consistency, and effect-size estimation of predicted biomarkers in control and co-culture groups. **(B)** LM1 PPI network analysis of DEPs in the STRING database using Cytoscape version 3.0. The nodes (proteins) and edges (combined scores) are plotted. Proteins reported to have adhesion or moonlighting ability (dark gray), putative function in adhesion (gray), and unknown adhesion capability (white). **(C)** Relative abundance of the top four discriminative proteins (EF-Tu/TufB, GAPDH, FuB, and EF-Ts) in LM1 after co-culture. *p*-values < 0.05 were considered significant.

The potential link between differentially expressed proteins (DEPs) and adhesion to IPEC-J2 cells was determined by LEfSe analysis. The top 20 discriminative LM1 proteins from the control included aldehyde dehydrogenase 2 (ALDH2), phosphoglycerate kinase 1 (PGK1), and histidine-binding periplasmic protein 1 (HisJ1) and 2 (HisJ2) (**Figure 2A**). In addition, several proteins including elongation factor Tu (EF-Tu/TufB), glyceraldehyde 3-phosphate dehydrogenase (GAPDH), phosphocarrier protein HPr (FuB), and elongation factor Ts (EF-Ts), had a higher LDA score in co-culture (**Figure 2B**). EF-Tu, GAPDH, and FuB are moonlighting proteins that exhibit multi-functionality, particularly in cell adhesion, and protect against bile stress (Dhanani and Bagchi, 2013; Lee et al., 2013; Kainulainen and Korhonen, 2014; Derkaoui et al., 2016). These moonlighting proteins have also been found on the cell surface of LM1 (unpublished data), providing further evidence of the correlation between these cytoplasmic proteins and their bacterial adhesion activity. In addition, some adhesion proteins with putative cell wall binding motifs, such as endo-beta-*N*-acetylglucosaminidase (LBLM1_00270), platelet binding protein GspB (LBLM1_04260 and LBLM1_04300), ATP-binding cassette transporter binding protein (LBLM1_04890), and the AAA ATPase containing von Willebrand factor type A (vWA) domains were found to be upregulated in co-culture conditions. In particular, ATP-binding cassette transporter binding protein is highly similar to the adhesin-like protein in *L. mucosae* ME-340 (Watanabe et al., 2010), which showed specific affinity to human blood group A and B antigens. In addition, proteins related to exopolysaccharide biosynthesis were decreased in mixed cultures (Supplementary Table S1), indicating again that LM1 adhesion to IPEC-J2 cells can be increased. Previous studies showed that the high adhesion ability of lactobacilli strains was inversely related to exopolysaccharide production (Denou et al., 2008; Polak-Berecka et al., 2014; Dertli et al., 2015).

Further analysis of these proteins using the STRING database revealed potential LM1 interacting proteins (**Figure 2C**). The PPI network showed a possible connection between LM1 proteins and bacterial adhesion. Several proteins were predicted to be co-expressed or interacting proteins based on the edges (lines) connecting the nodes (proteins). As mentioned above, EF-Tu, GAPDH, and FuB were originally identified as intracellular proteins, but have frequently been identified as adhesion factors. It is still unknown how these proteins are transported out of the cell since they lack signal peptides as a transport signal. Signal peptide recognition and transport proteins (SecA) and (Ffh) were found to be directly involved in the extracellular transport of many of these proteins including EF-Tu, GAPDH, and FuB, even though they lack a proper signal peptide (**Figure 2C**). Other than signal peptide-based transport, an alternative non-classical transport mechanism has been identified; however, it is unclear how transport proteins work when signal peptides are lacking, as observed in this study. Additional studies are required to elucidate the unique mechanism in this unconventional mode of intracellular protein transport. In addition, several other proteins should also be investigated for their potential link to LM1 adhesion or signaling ability. Cloning experiments should be performed to further characterize these proteins. This will reveal potential immunomodulatory properties and provide insight on their role in adhesion to IPEC-J2 cells.

### Proteomic Profile of IPEC-J2 Cells in Co-culture Conditions

**FIGURE 3:**
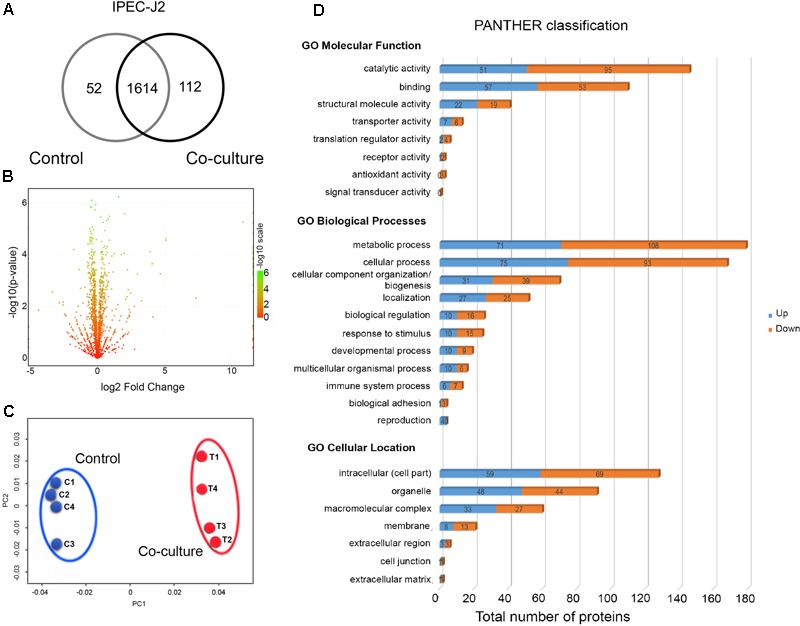
Global proteome profile of IPEC-J2 cells in co-culture conditions. **(A)** Venn diagram of unique and shared proteins detected in IPEC-J2 cells. **(B)** A volcano plot of the deactivated/downregulated (left, 376) and activated/upregulated (right, 277) proteins in IPEC-J2 cells. *p*-values were calculated and plotted in the *y*-axis as –log10(*p*-value). Values for *y*-axis [–log10(*p*-value)] above 1.3 were considered significant. **(C)** Non-metric multidimensional scaling (NMDS) plot of individual IPEC-J2 proteomes showing distinct clustering of each group. Individual proteomes are separated in control (C1–C4) and co-culture (T1–T4). **(D)** Functional classification of LM1 proteins using Gene Ontology (GO) classification from the PANTHER database (Molecular Function, Biological Processes, and Cellular Location). The numbers of downregulated (orange) and upregulated (blue) proteins are shown.

We compared the proteomes of IPEC-J2 cells grown in control and in co-culture with LM1. The co-culture experiment demonstrated mutualism between IECs and intestinal bacteria. Intracellular proteins from IPEC-J2 were identified and quantified and totals of 1666 and 1726 proteins were detected in control and co-culture conditions, respectively (**Figure 3A**). Among them, 93% of proteins (1614) were present in both conditions, while 52 and 112 proteins were uniquely identified in control and co-culture, respectively (**Figure 3A**).

Among the detected proteins, 270 proteins (15.2%) were significantly upregulated (*p* < 0.05) with up to a 34.5-fold increase, while 369 proteins (20.7%) were significantly downregulated with up to a 0.04-fold reduction after co-culture with LM1 (**Figure 3B** and Supplementary Table S2). A comparison of global proteomic profiles between control and co-culture revealed a distinct separation between the two groups (**Figure 3C**), suggesting that co-culture with LM1 influenced the biological activity and cellular processes of IPEC-J2 cells.

We further analyzed DEPs using the PANTHER GO database and they grouped independently into three major categories: (a) Molecular Function, (b) Biological Processes, and (c) Cellular Location. Under the Molecular Function category, the majority of the proteins were involved in catalytic activity, binding, and structural molecule activity. However, under the Biological Processes category, the majority of the DEPs were involved in metabolic processes, cellular processes, and cellular component organization and biogenesis. After the co-culture experiment, the majority of the proteins were primarily located in the intracellular region, followed by organelles and macromolecular complexes (**Figure 3D**). Based on these results, IPEC-J2 metabolic, cellular, and structural properties undergo significant modifications in response to LM1 establishment, cellular reorganization, and protein localization.

### IPEC-J2 Functional Proteins and Pathways Influenced by *L. mucosae* LM1

**FIGURE 4:**
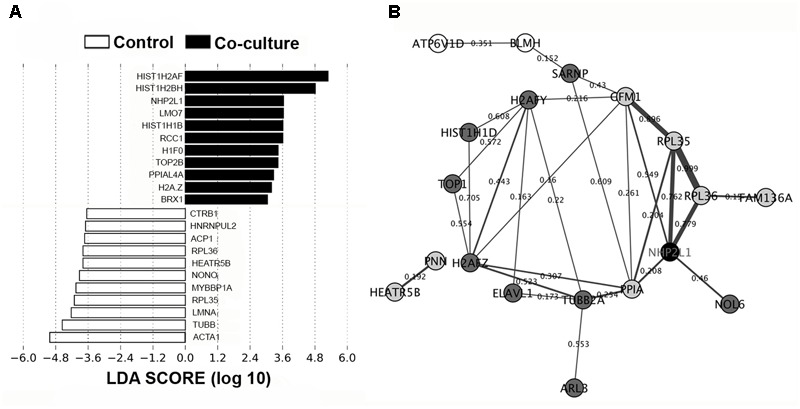
Biomarker analysis and PPI network in the IPEC-J2 proteome. **(A)** LEfSe score showing the statistical significance, biological consistency, and effect-size estimation of predicted biomarkers in control and co-culture groups. **(B)** IPEC-J2 PPI network analysis of DEPs in the STRING database using Cytoscape version 3.0. The nodes (proteins) and edges (combined scores) are plotted. Proteins that were activated (black), upregulated (dark gray), downregulated (light gray), and deactivated (white). Co-expression values are represented by the thickness of the edges (thicker edges – high co-expression, thinner edges – low co-expression).

Using LEfSe analysis, we identified potential protein biomarkers in control and co-culture groups (**Figure 4A**). Analysis of the composition of proteomic profiles using LEfSe analysis showed that α1 skeletal muscle (ACTA1), tubulin β class I (TUBB), lamin A/C (LMNA), ribosomal protein L35 (RPL35), and myoglobin-binding protein 1A (MYBBP1A), etc, were the most prominent in the IPEC-J2 cells without co-incubation with *L. mucosae* LM1. On the other hand, histone proteins (HIST1H2AF, HIST1H2BH, HIST1H1B, H1F0, and H2A.Z), non-histone chromosome protein 2-like 1 (NHP2L1), LIM domain only protein 7 (LMO7), regulator of chromosome condensation (RCC1), DNA topoisomerase 2-beta (TOP2B), PPIAL4A, and ribosomal biogenesis protein (BRX1) were highly associated in the IPEC-J2 cells after co-incubation with LM1. These results suggest that cell structure and integrity is a vital feature of IECs in normal condition, whereas proteins associated with regulation of gene expression highly up-regulated by LM1.

In addition, we generated a potential PPI network using a STRING database from DEPs from IPEC-J2 cells to display how IECs may regulate these proteins. **Figure 4B** shows that the activation of NHP2L1 is involved in ribosomal function (RPL35 and RPL36) and protein phosphorylation (PPIA), which play roles in tight junction (TJ) formation and signaling. Several proteins associated with regulating gene expression were also observed in the network. In particular, histones including H2A type 1-F (34.5-fold) and 1-H (34.3-fold), H1.5 (25.5-fold), H2AFZ (17.5-fold), H2AFY (7.7-fold), HIST1H1D (5.1-fold), and DNA topoisomerase 1 (TOP1, 2.8-fold) were found in the network.

To further investigate whether LM1 adhesion impacts the overall function of IPEC-J2 cells, we classified 456 out of 653 DEPs into 240 pathways in the KEGG database (Supplementary Table S3). LM1 influenced many pathways in IPEC-J2 cells, including TJ formation, actin cytoskeleton regulation, PI3K-Akt signaling, gap junction formation, apoptosis, antigen processing and presentation, and bacterial invasion of epithelial cells. Furthermore, KEGG analysis showed that 16 and 12 proteins that were up-regulated after co-incubation with LM1 were involved in the assembly of TJs and the actin cytoskeleton, respectively. These pathways are essential for bacterial establishment and cell integrity, which protect against toxins and pathogens (Mounier and Arrigo, 2002; Candela et al., 2008; Anderson et al., 2013; Liu et al., 2015; Yang et al., 2015; Yu et al., 2015).

The results indicate that probiotic bacteria may significantly affect the cellular physiology of IECs. However, at this point, only a number limited of studies have been able to demonstrate how probiotic *Lactobacillus* strains modulate TJ assembly, histone levels, and cell signaling pathways of IECs.

### Correlational Network Analysis of LM1 and IPEC-J2 Proteins

**FIGURE 5:**
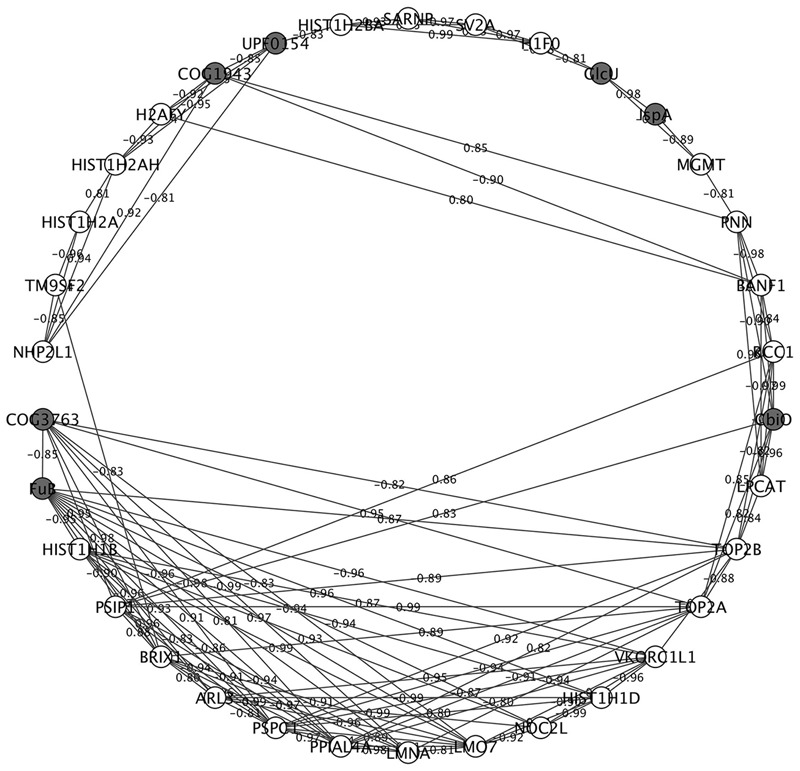
Correlational network analysis of LM1 and IPEC-J2 proteins. Cytoscape visualization shows the high association between the nodes (proteins) and edges (correlation coefficient). Negatively and positively correlated proteins are indicated in the edges of each interaction. Proteins from LM1 (dark gray) and IPEC-J2 (white) proteomes are indicated. The absolute correlation coefficient of 0.8 or higher of highly regulated proteins was used in the plot.

We constructed an interaction network of LM1 and IPEC-J2 proteins based on a correlation matrix (**Figure 5**). The matrix was created from significantly overexpressed LM1 and IPEC-J2 proteins with an absolute fold-change value greater than 4.0. A high correlation value was used as the threshold (0.8) for significant correlation (*p* < 0.05). In total, 34 nodes (seven LM1 and 27 IPEC-J2 proteins) were plotted. LM1 proteins displayed significant correlation with IPEC-J2 proteins including REP element-mobilizing transposase RayT (COG1943), unknown protein function (UPF0154), transmembrane sugar transporter (GlcU), geranyltranstransferase (IspA), energy-coupling factor transporter ATPase (CbiO), FuB, and uncharacterized protein YneF (COG3763). Four out of seven LM1 proteins were activated in the presence of intestinal cells, indicating a unique signature for host–microbe crosstalk. These proteins (GlcU, IspA, CbiO, and UPF0154) were linked to IPEC-J2 proteins that are responsible for the regulation of gene expression via histones (i.e., HIST1H2AH, H2AFY, HIST1H2BA, and H1F0) or non-histone proteins (i.e., NHP2L1). Furthermore, FuB was positively correlated with TOP2B and HIST1H1D; this may indicate signaling capability to regulate gene expression in intestinal cells. However, these proteins must be further investigated for their potential ability to immunomodulate the host via interaction with IECs.

## Discussion

**FIGURE 6:**
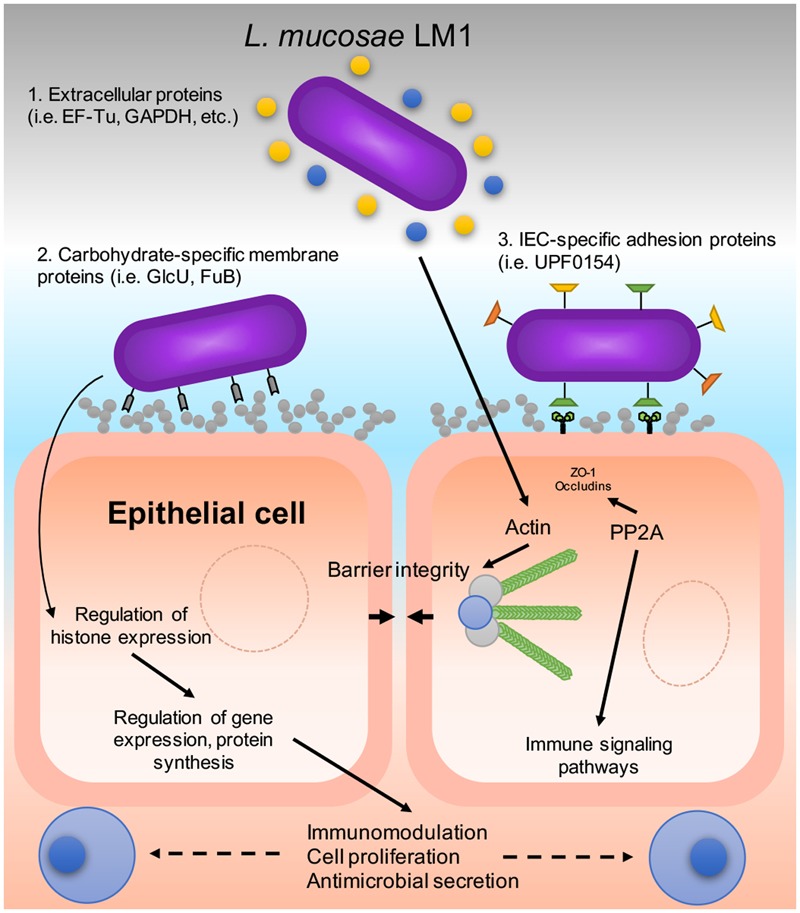
A proposed host–microbe interaction model of LM1 and IPEC-J2 cells. The diagram shows the mechanism of adhesion that could be exhibited by LM1 in various ways: (1) Moonlighting proteins that are secreted externally via the bacterial secretion system, (2) carbohydrate-specific binding proteins, and (3) intestinal epithelial cell (IEC)-specific adhesion proteins. LM1 establishment can influence IEC function by regulating tight junction (TJ) and barrier integrity via TJ dephosphorylation and actin regulation. Histone levels and post-translational mechanisms are also influenced by LM1 that may lead to gene regulation, the secretion of host antimicrobial compounds, and immunomodulation.

Deciphering the molecular basis of host–microbe interaction is crucial for understanding the probiotic mechanism of lactobacilli and how the bacteria can avoid instantaneous peristaltic exclusion for its establishment and colonization on the intestinal mucosa. Here, we used the potential probiotic *L. mucosae* LM1 as a model to investigate communication between probiotic lactic acid bacteria and IECs because the LM1 strain was reported to be highly adhesive to IPEC-J2 cells (Valeriano et al., 2014, 2016). In order to mimick the transit time of the bacteria in the gastrointestinal tract, we co-incubated *L. mucosae* LM1 and IPEC-J2 for 1 h prior to cell harvest. However, the precise mechanism of adhesion to commensal bacteria, such as lactobacilli, is not known. In this study, the functional activities of LM1, and its ability to stimulate the intestinal environment (IPEC-J2) and vice versa, allowed for the identification of proteomic signatures associated with host–microbe interaction (**Figure 6**). Differentially regulated proteins may provide valuable insight into bacterial colonization, immune response regulation, and gastrointestinal functions that are influenced during their establishment. Clearly, co-incubation altered the global protein profiles in both the bacteria and host intestinal cells, suggesting that their biological and molecular functions were significantly changed by cell–cell interaction.

After co-incubation with IPEC-J2 cells, the top three DEPs in *L. mucosae* LM1 were EF-Tu/TufB, GAPDH, and FuB. It indicates that IPEC-J2 cells may induce the expression of these proteins in LM1, then confer higher adhesion capability of LM1 because these proteins were also observed in other adherent *Lactobacillus* species (Kinoshita et al., 2008a; Dhanani and Bagchi, 2013; Kainulainen and Korhonen, 2014). These proteins, originally found in the cytoplasm, were also identified to be moonlighting proteins, which are found extracellularly and can mediate bacterial establishment to IECs (Granato et al., 2004; Zhang et al., 2015; Derkaoui et al., 2016). In a previous study, it was shown that EF-Tu on the surface of *L. johnsonii* La1 improved binding to intestinal cells and mucins (Granato et al., 2004), which might also participate in gut homeostasis by binding with the intestinal epithelia and maintaining barrier structure and integrity. GAPDH also facilitated the binding of several strains of lactobacilli to colonic Caco-2 cells and human ABO-blood type antigens (Kinoshita et al., 2008b; Zhang et al., 2015). In addition, GAPDH may also possess antigenic and immunomodulatory properties that has the potential to induce B and T cell activation or increase IL-10 production in the host (Kainulainen and Korhonen, 2014). Thus, regardless of the type of intestinal cell, these proteins can facilitate and mediate bacterial adhesion to intestinal epithelia. The factors of the epithelial cells responsible for the induction of LM1 protein expression should be identified in the future.

LM1 activated other major IPEC-J2 proteins including regulator of chromosome condensation (RCC1), DNA-topoisomerase 2-β (TOP2B), histone H1.0 (H1F0), ribosome biogenesis protein BRX1 (BRX1), and NHP2L1 proteins. LM1 also caused a >5.1-fold increase in different types of histones; this indicates a significant alteration in gene expression and protein biosynthesis in IPEC-J2 cells. There are several families and subtypes of histones, which are ubiquitously expressed (Boyle, 2005). H1 histones (H1.0 and H1.5), also called linker histones, stabilize compact, higher order structures of chromatin (Warneboldt et al., 2008). On the other hand, H2A and subtypes (1-F and 1-H) are core histones involved in packaging DNA into chromatin (Mariño-Ramírez et al., 2006). In addition to their role as structural proteins, histones also actively regulate gene expression and participate in chromatin-based processes like DNA replication and repair. The epigenetic contribution of H1 and H2A histones to these mechanisms makes it conceivable that they also have roles in cell proliferation, differentiation, and the inflammatory response. Furthermore, it has been reported that histone H2A can affect the host immune response by acting as an antimicrobial peptide (AMP). Histone deacetylase 2 (HDAC2) was also upregulated by LM1 (Supplementary Table S2). It has been reported that epithelial HDAC2 restrains intestinal inflammation by regulating IEC proliferation and differentiation (Turgeon et al., 2013). Gonneaud et al. (2016) suggested that HDAC2 is directly involved in IEC determination and intestinal homeostasis, and that changes in the IEC acetylome may alter the mucosal environment. The acetylation and deacetylation of histones and other proteins is dependent on the activities of histone acetyltransferases and histone deacetylases (HDACs), which mediate the regulation of gene expression. Future studies on the roles of specific histones and HDACs in the intestinal environment will provide more information and help elucidate their exact function in gut immunity.

The expression of TJ proteins in intestinal cells was also affected by LM1. Here, the TJ proteins, zonula occludens (ZO)-1, ZO-2 and occludins were down-regulated slightly by LM1. TJ proteins are important for bacterial establishment and IEC integrity (Yang et al., 2015; Yu et al., 2015). They act as a primary defense against bacterial invasion. However, slight down-regulation of TJ proteins changes cell permeability and then might facilitate trans-epithelial passage of LM1. However, its relationship with bacterial adhesion cannot be excluded, which needs to be elucidated through further experiments.

In addition to the downregulation of TJ proteins, a significant upregulation of protein phosphatase 2A (PP2A) was detected after co-incubation with LM1. PP2A is a major Ser/Thr protein phosphatase that is linked to the localization of TJ proteins in the membrane, particularly ZO-1, occludins, and claudins (Seth et al., 2007; Rao and Samak, 2013). In addition, PP2A and its many subtypes (i.e., PPP2R1A) are important for intracellular signaling since PP2A is associated with the dephosphorylation of Akt and mitogen-activated protein kinase kinase (MEK). Akt and MEK are integral members of the PI3K-Akt-mTOR signaling and mitogen-activated protein kinase (MAPK) signaling cascades, respectively, and are both important for cell proliferation and apoptosis (Zimmerman et al., 2012; Wlodarchak and Xing, 2016). BCL-2-like protein (BCL2L13), an anti-apoptotic protein, and insulin-like growth factor-binding protein 3 (IGFBP3) were also upregulated, whereas cytochrome c oxidase (COX5B) and toll-like receptor 9 (TLR9) were downregulated; therefore, cell survival and proliferation may be beneficial effects induced by LM1. In addition, PP2A has been identified as a biomarker or drug target for various gastrointestinal and neurological disorders (Watkins et al., 2012; Toda-Ishii et al., 2016; Nematullah et al., 2017).

## Conclusion

In this study, we identified and quantified, for the first time, the global proteins that might be involved in interaction between probiotic *L. mucosae* LM1 and the porcine intestinal epithelia. A number of well-known moonlighting proteins and newly identified proteins might be associated with adhesion ability of LM1. In addition, proteins that are involved in cell structure and gene regulation in intestinal cells are regulated by LM1. These proteins can act as biomarkers for bacterial cell-to-intestinal cell signaling. Further studies are needed to elucidate how IECs discriminate and respond to intestinal bacteria with different genomic and functional profiles.

## Author Contributions

D-KK designed this study. EP, SK, VV, and JL performed the experiments. EP and D-KK wrote and revised the manuscript. All authors approved the final manuscript to be published.

## Conflict of Interest Statement

The authors declare that the research was conducted in the absence of any commercial or financial relationships that could be construed as a potential conflict of interest.

## Footnotes

1 http://www.string-db.org

2 http://proteomecentral.proteomexchange.org

## Supplementary Material

FIGURE S1 Experimental design of co-culture and global proteome analysis using Orbitrap mass spectrometry (MS). A 1-h co-culture of *L. mucosae* LM1 (LM1) and porcine intestinal epithelial cells from neonatal jejunum (IPEC-J2). The label-free proteomic analysis was performed using Orbitrap MS.

## Abbreviations

ANOVA: analysis of variance
COG: cluster of orthologous groups
GO: Gene Ontology
HESI-II: heated electrospray ionization source
IECs: intestinal epithelial cells
IPEC-J2: intestinal porcine epithelial cell line
KEGG: Kyoto Encyclopedia of Genes and Genomes
LDA: Linear discriminant analysis
LEfSe: LDA effect size
LM1: *Lactobacillus mucosae* LM1
MRS: de Mann, Rogosa, and Sharpe
MS: mass spectrometry
PPI: protein–protein interaction network
