# Supplementary figures and images for: Proteomic View of the Crosstalk between Lactobacillus mucosae and Intestinal Epithelial Cells in Co-culture Revealed by Q Exactive-Based Quantitative Proteomics
Source: Front Microbiol. 2017 Dec 12;8:2459. doi: 10.3389/fmicb.2017.02459 (PMC5732961; doi:10.3389/fmicb.2017.02459)

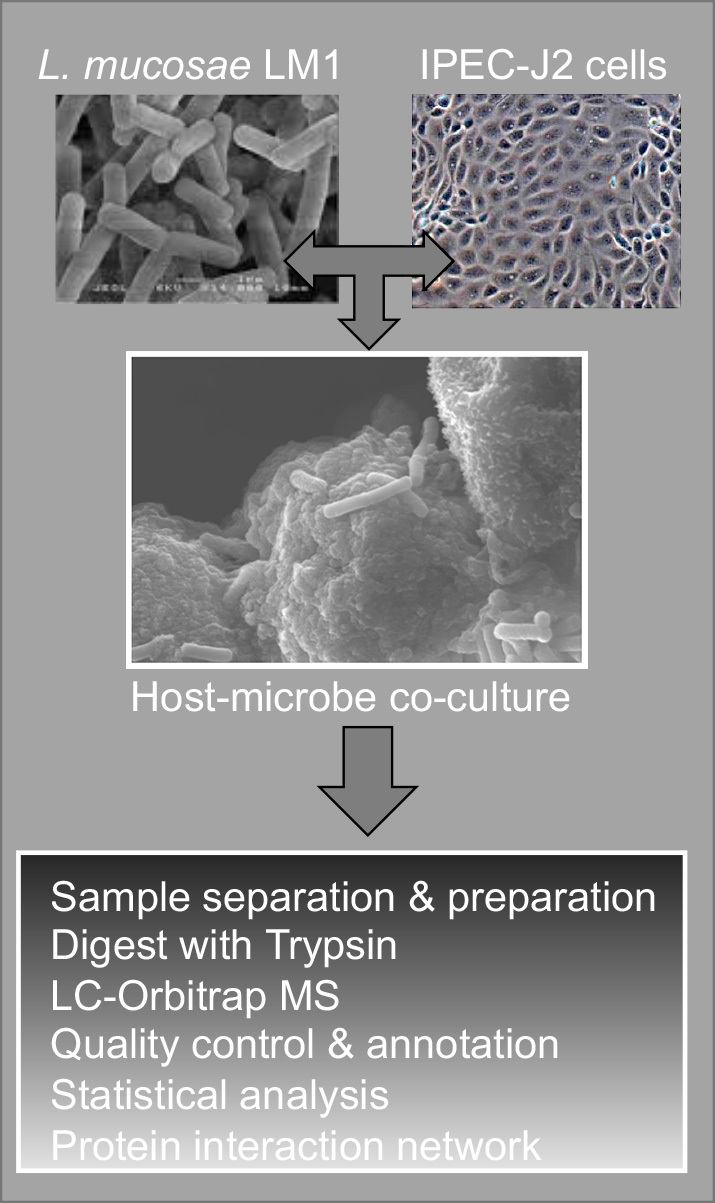

Supplement: FIGURE S1 — Experimental design of co-culture and global proteome analysis using Orbitrap mass spectrometry (MS). A 1-h co-culture of L. mucosae LM1 (LM1) and porcine intestinal epithelial cells from neonatal jejunum (IPEC-J2). The label-free proteomic analysis was performed using Orbitrap MS. [file Image_1.JPEG]
